# Characterization of the Fifth Putative Acetylcholinesterase in the Wolf Spider, *Pardosa pseudoannulata*

**DOI:** 10.3390/molecules22071118

**Published:** 2017-07-11

**Authors:** Xiangkun Meng, Xixia Xu, Haibo Bao, Jianjun Wang, Zewen Liu

**Affiliations:** 1 Key Laboratory of Integrated Management of Crop Diseases and Pests (Ministry of Education), College of Plant Protection, Nanjing Agricultural University, Weigang 1, Nanjing 210095, China; mxk@yzu.edu.cn (X.M.); 2016102079@njau.edu.cn (X.X.); baohaibo2014@126.com (H.B.); 2 College of Horticulture and Plant Protection, Yangzhou University, Yangzhou 225009, China; wangjj@yzu.edu.cn

**Keywords:** *Pardosa pseudoannulata*, acetylcholinesterase (AChE), sensitivity

## Abstract

*Background:* Acetylcholinesterase (AChE) is an important neurotransmitter hydrolase in invertebrate and vertebrate nervous systems. The number of AChEs is various among invertebrate species, with different functions including the ‘classical’ role in terminating synaptic transmission and other ‘non-classical’ roles. *Methods*: Using rapid amplification of cDNA ends (RACE) technology, a new putative AChE-encoding gene was cloned from *Pardosa pseudoannulata*, an important predatory natural enemy. Sequence analysis and in vitro expression were employed to determine the structural features and biochemical properties of this putative AChE. *Results:* The cloned AChE contained the most conserved motifs of AChEs family and was clearly clustered with Arachnida AChEs. Determination of biochemical properties revealed that the recombinant enzyme had the obvious preference for the substrate ATC (acetylthiocholine iodide) versus BTC (butyrylthiocholine iodide). The AChE was highly sensitive to AChE-specific inhibitor BW284C51, but not butyrylcholinesterase-specific inhibitor tetraisopropyl pyrophosphoramide (ISO-OMPA). Based on these results, we concluded that a new AChE was identified from *P. pseudoannulata* and denoted as PpAChE5.* Conclusion:* Here we report the identification of a new AChE from *P. pseudoannulata* and increased the AChE number to five in this species. Although PpAChE5 had the biggest *V*_max_ value among five identified AChEs, it showed relatively low affinity with ATC. Similar sensitivity to test insecticides indicated that this AChE might serve as the target for both organophosphorus and carbamate insecticides.

## 1. Introduction

As a dominating neurotransmitter hydrolase, acetylcholinesterases (AChEs) extensively exist in invertebrate and vertebrate nervous systems, and effectively hydrolyse acetylcholine to maintain the normal nerve conduction. The importance of AChEs makes it a research hotspot in human medical treatments and agricultural pest management [1,2,3,4,5]. Besides the classical synaptic hydrolytic function, a variety of AChEs non-cholinergic functions have been found [6,7,8]. AChEs may have multiple non-cholinergic functions in organisms, and different AChEs in one species may have various physiological functions [9]. More direct evidences were needed to illuminate the overall functions of AChEs.

AChEs are encoded by *ace* genes. The generation of distinct multiple AChE isoforms may occur via *ace* gene duplications and alternative splicing, and then different structural and functional AChEs are generated [10]. The number of AChEs varies among species, such as one or two AChEs in insects, four different AChEs in nematodes [10,11]. In our previous study, four AChEs (PpAChE1-4) possessing diverse biochemical properties were identified from an important natural enemy spider *Pardosa pseudoannulata*. However, additional AChEs may exist since multiple unigenes were annotated as *ace* in this spider transcriptome [12,13]. So, it is interesting to elucidate the physiological functions of each AChE and their involvement in insecticide sensitivity, because *P. pseudoannulata* are often exposed to insecticides targeting insect pests.

Based on the *P. pseudoannulata* transcriptome and the previous identification of four AChEs, we reported the fifth AChE (PpAChE5) in *P. pseudoannulata* in this study. Amino acid sequence characteristics and the biochemical properties of PpAChE5 were analyzed and compared with PpAChE1-4. Our results provide important information for the understanding of the structural differentiation that influence enzyme properties, and offer basic research for the study of AChEs functions.

## 2. Results

### 2.1. Cloning and Sequence Analysis of the Fifth Putative Ace Gene from P. pseudoannulata

**Figure 1:**
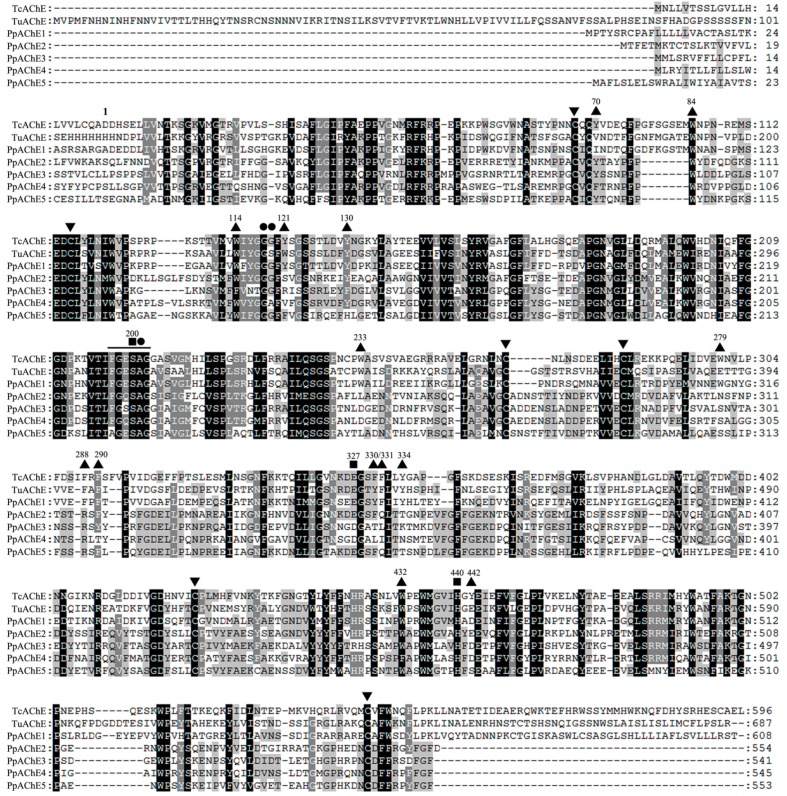
Amino acid sequence alignment of acetylcholinesterase (AChEs) from *P. pseudoannulata* and other species. Identical amino acids are shaded in black for 100% identity and grey for 80% similarity. The ‘▲’ represents the 14 aromatic residues, ‘▼’ indicates the six cysteine residues, ‘■’ shows the catalytic triads, and ‘●’ indicates the oxyanion hole. The conserved sequence ‘FGESAG’ is underlined. The numbering on the amino acid sequences indicates the positions for *Torpedo californica* AChE amino acids, which starts at the N-terminus of the mature protein. Tc: *Torpedo californica* (CAA27169); Tu: *Tetranychus urticae* (AAO73450); Pp: *Pardosa pseudoannulata* (KF543247, KU501286, KU501287, KU501288, KU501289).

In addition to four AChEs (PpAChE1-4) we identified from *P. pseudoannulata*, one new putative *ace* gene was found in *P. pseudoannulata* transcriptome and was confirmed by polymerase chain reaction. The full-length cDNA (GenBank Accession number: KU501289) was obtained by RACE technology, which has an open reading frame of 1662 bp. The deduced amino acid sequence (553 in length) shows high identity to PpAChE2-4 (42.2–48.3%), and is 24.6–28.3% identical in pairwise comparisons with PpAChE1 and *Torpedo californica* and *Tetranychus urticae* AChEs (Figure 1). Based on the sequence similarity, the new putative AChE was named PpAChE5.

**Figure 2:**
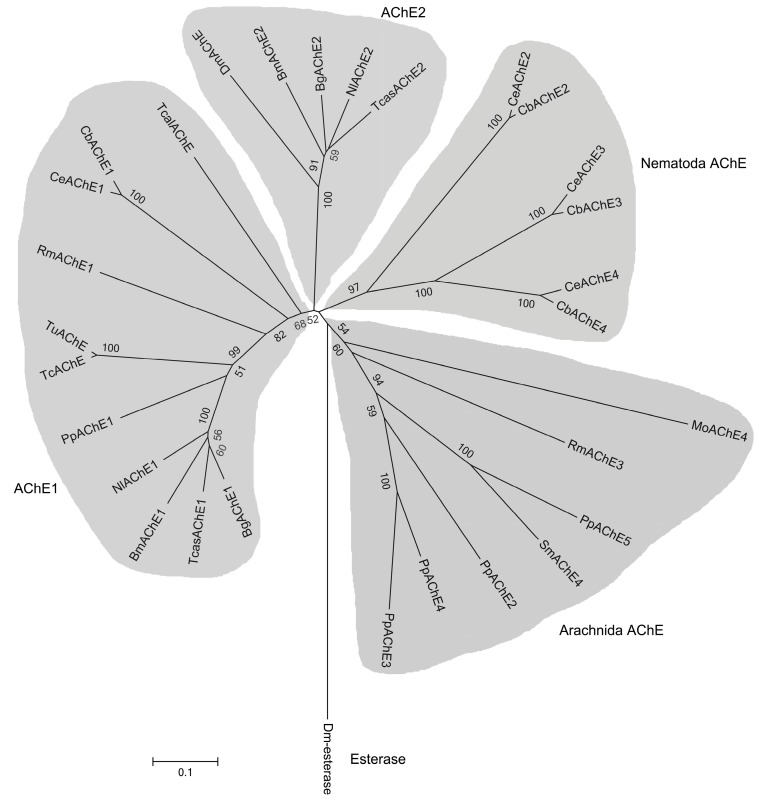
Phylogenetic analysis of PpAChE5 compared with AChEs from *P. pseudoannulata* and other species. Numbers above the branches indicate phylogenies based on amino acid sequences, and only values above 50% are shown. Tcal: *Torpedo californica* (TcalAChE: CAA27169); Dm: *Drosophila melanogaster* (DmAChE: P07140; Dm-esterase: AAP21002); Bg: *Blattella germanica* (BgAChE1: ABB89946; BgAChE2: ABB89947); Bm: *Bombyx mori* (BmAChE1: ABB05341; BmAChE2: ABY50089); Nl: *Nilaparvata lugens* (NlAChE1: ADZ15146; NlAChE2: AFC61184); Tcas: *Tribolium castaneum* (TcasAChE1: ADU33189; TcasAChE2: ADU33190); Tc: *Tetranychus cinnabarinus* (TcAChE: AGI96546); Tu: *Tetranychus urticae* (TuAChE: ADK12702); Rm: *Rhipicephalus microplus* (RmAChE1: AJA71270; RmAChE3: ALD51323); Ce: *Caenorhabditis elegans* (CeAChE1: X75331; CeAChE2: AF025378; CeAChE3: AF039650; CeAChE4: AF025379); Cb: *Caenorhabditis briggsae* (CbAChE1: U41846; CbAChE2: AF030037; CbAChE3: AF159504; CbAChE4: AF159505); Mo: *Metaseiulus occidentalis* (MoAChE4: XP_003739938); Sm: *Stegodyphus mimosarum* (SmAChE4: KFM73382); Pp: *Pardosa pseudoannulata* (PpAChE1: KF543247; PpAChE2: KU501286; PpAChE3: KU501287; PpAChE4: KU501288; PpAChE5: KU501289).

Phylogenetic tree of PpAChE5 with PpAChE1-4 and AChEs from other species was constructed, and it clearly showed that PpAChE5 has a relatively close evolutionary relationship with Arachnida AChEs including PpAChE2-4, but not PpAChE1 (Figure 2). Amino acid sequence alignment shows that PpAChE5 has most structure characteristics of AChEs family including the ‘SEH’ catalytic triad, conserved cysteine residues and choline binding sites (Figure 1). However, some amino acids which were important for AChE functions were different among PpAChE5 and other AChEs, such as the conserved sequence ‘FGESAG’ and aromatic residues (Table 1).

### 2.2. Recombinant Expression and Enzyme Activity Assay

Using Bac-to-Bac systems, PpAChE5 and the enhanced green fluorescent protein (EGFP) were expressed in Sf9 cells. The detection of fluorescence in EGFP infectious cells and virus-infected cell form in both protein expressive cells indicated the successful recombinant expression (data not show). Baculovirus culture supernatants which included the expressed proteins were collected for further study.

**Figure 3:**
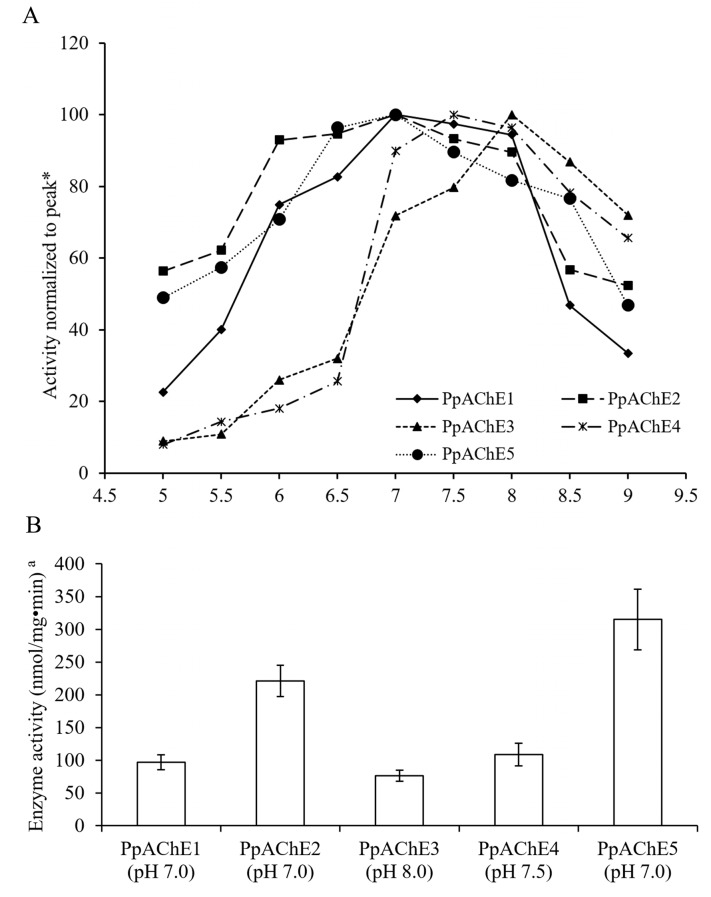
Optimal pH conditions for PpAChE5. (**A**): Enzyme activities under different pH conditions. (**B**): Enzyme activities of five AChEs under their optimal pH conditions. Data are the mean ± SEM of at least five independent experiments. For clarity, only one representative curve for each enzyme is shown. Data of PpAChE1-4 were from our previous study [12].

Enzyme activities of the expressed PpAChE5 were measured at different times after the virus infection, and the highest activity was observed at 72 h after cell infection, which was identical to our previous study [12]. Activities of PpAChE5 under various pH conditions were then determined. The result showed that PpAChE5 has the maximum activity (315.18 nmol/mg·min) at pH 7.0, which was much higher than the enzyme activities of PpAChE1-4 (Figure 3) [12].

### 2.3. Substrate Hydrolysis Kinetics of PpAChE5

To identify the substrate preference of PpAChE5, three substrates, acetylthiocholine iodide (ATC), butyrylthiocholine iodide (BTC) and propionylthiocholine iodide (PTC), were used to analyze the kinetic parameters including Michaelis–Menten constant value (*K*_M_) and maximal reaction velocity (*V*_max_) (Table 2). The results showed that PpAChE5 had similar *K*_M_ values for all three substrates. However, the *V*_max_ values of PpAChE5 for the substrate ATC was 4.6 and 7.2 times of that for the BTC and PTC, respectively. Similarly, PpAChE5 showed the highest catalytic efficiency (*V*_max_/*K*_M_) for the substrate ATC, which was about 5.4 and 8.6 times of that for BTC and PTC respectively. The comparison of *V*_max_ ratios also indicated that PpAChE5 has a preference for substrate ATC (Table 2).

### 2.4. Inhibition Kinetics of PpAChE5

**Figure 4:**
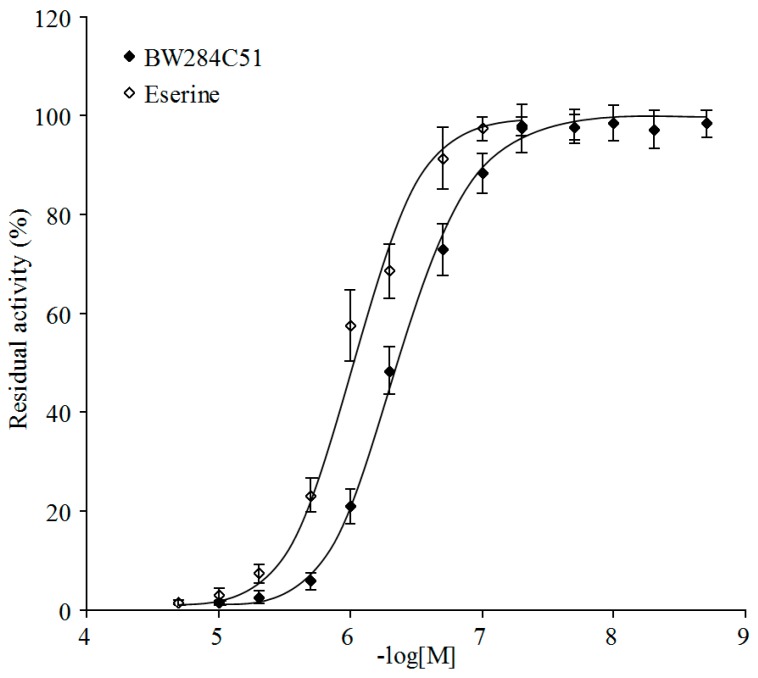
Inhibition curves of two inhibitors against ATC hydrolysis activity of PpAChE5. The data are the mean ± SEM of at least five independent experiments.

Three cholinesterase inhibitors, the cholinesterase-specific inhibitor eserine, acetylcholinesterase-specific inhibitor 1,5-bis(4-allyldimethylammoniumphenyl)-pentan-3-one dibromide (BW284C51), and butyrylcholinesterase-specific inhibitor tetraisopropyl pyrophosphoramide (ISO-OMPA), were used to determine their inhibition on PpAChE5 enzyme activity (Table 3). Both BW284C51 and eserine showed the strong inhibition on ATC hydrolysis activity of PpAChE5, indicating that PpAChE5 was sensitive to acetylcholinesterase-specific inhibitors (Figure 4). In contrast, ISO-OMPA showed very weak inhibition against BTC hydrolysis with the *IC*_50_ value above 0.2 mM, suggesting that PpAChE5 was not sensitive to butyrylcholinesterase-specific inhibitors.

Further study showed that the ATC hydrolysis activity of PpAChE5 was also inhibited by two organophosphorus insecticides (paraoxon and diazoxon) and two carbamate insecticides (fenobucarb and carbaryl), and with similar sensitivities (Table 4).

## 3. Discussion

AChEs have been widely studied in invertebrates and vertebrates, but the numbers and overall functions of AChEs in some species remain undefined. In the present study, in addition to four AChEs (PpAChE1-4) previously identified from *P. pseudoannulata*, we reported the identification of a new AChE (PpAChE5) in this species [12]. Sequence alignment showed that PpAChE5 possessed most of the crucial structure features of AChEs family, including the important choline binding sites, catalytic triads functioning as a charge-relay system, a number of aromatic residues lining the catalytic gorge and six cysteine residues forming three intramolecular disulphide bridges. These conserved amino acids have been reported to play important roles in AChE functions [14]. However, this putative AChE also has some unique features, such as the ‘AGESAG’ instead of ‘FGESAG’ and only seven out of fourteen aromatic residues are conserved. Among the five AChEs of *P. pseudoannulata*, PpAChE5 showed more than 40% identities to PpAChE2-4, and many important amino acids among these four PpAChEs are identical. Phylogenetic analysis of PpAChE1-5 with other species AChEs also showed that PpAChE2-5 were closely related to each other, indicating that they might have evolved from a common orthologous gene.

Although PpAChE5 showed high identities to PpAChE2-4, their biochemical properties are significantly different, such as the optimal pH, *K*_M_, *V*_max_ and inhibitor sensitivities. The *K*_M_ values of PpAChE5 to three substrates were similar and significantly lower than that of PpAChE3 and PpAChE4. However, PpAChE5 displayed the highest *V*_max_ value against the substrate ATC among five PpAChEs. PpAChE5 also displayed the selectivity among three substrates, with higher catalytic efficiency (*V*_max_*/K*_M_) for ATC than that for BTC and PTC. In our previously studies, PpAChE1-4 showed significantly different sensitivities to organophosphorus and carbamate insecticides in vivo and in vitro [12,15,16]. However, PpAChE5 showed similar sensitivities to four tested insecticides in this study.

Most insect species have two AChEs with different structures and biochemical properties, and a few insects only have one AChE, such as *Drosophila melanogaster* and *Musca domestica* [10,17,18]. The only AChE in model insect *D. melanogaster* shared high identities with insect AChE2 and generated multiple molecular forms via alternative splicing, such as membrane-anchored and soluble forms, and had different expression patterns as well as catalytic activities [10]. The generation of distinct multiple AChE2 isoforms via alternative splicing suggests that the functional diversification of AChE2 can be obtained, which may have allowed the loss of *ace1* during the process of functional replacement of AChE1 with AChE2 [10]. Multiple AChEs with different biochemical properties in one species have been identified, such as nematodes, ticks, insects and spiders, and these enzyme property differences may contribute to different physiological functions [10,11,19]. In addition to the classical enzymatic catalytic function, the non-classical functions have been determined in mammal, including neuritogenesis and synaptogenesis [20,21,22], cell adhesion and apoptosis [23,24], activation of dopamine neurons [25], amyloid fibre assembly [26], haematopoiesis and thrombopoiesis [27,28], inflammation and immunoreaction [29,30]. However, some of the above-proposed non-classical functions are based merely on correlations and circumstantial evidences, and some might not yet be disproved [9]. The non-classical functions should also exist in insects with only one or two AChEs, although these functions were rarely studied and primarily focus on insect growth and development [31,32,33,34]. Suppressing the expression of *Helicoverpa armigera ace* through RNA interference resulted in the growth inhibition of larvae, reduction in pupal weight, malformation and reduced fecundity [35]. In insects with two AChEs, the effects of knockdown of *ace1* or *ace2* on insect growth and development were different. Silencing *Tribolium castaneum ace1* prominently increased the mortality and insecticide susceptibility, whereas silencing *ace2* delayed insect development and reduced female egg-laying and egg-hatching [31]. In *Rhopalosiphum padi* and *Sitobion avenae*, the suppression of *ace1* increased susceptibilities to insecticides and also caused significant reductions in fecundity, whereas knockdown of *ace2* only had a slight effect on their susceptibilities [32]. In lepidoptera insect *Chilo suppressalis*, knockdown of *ace1* or *ace2* caused a 25% mortality rate, and silencing *ace1* dramatically inhibited larval growth and reduced larval weight and length, malformation, whereas silencing *ace2* merely had minor effects [33]. One recent study also found that the expression of *Apis mellifera* AChE1 is associated with brood rearing status [34]. In this study, the fifth AChE was identified from *P. pseudoannulata* and makes this spider become the species containing the maximum number of AChEs so far. Functions of each specific PpAChE were needed to be interpreted, especially for the non-classical functions. 

In summary, the present study reported the identification of the fifth AChE from the natural enemy *P. pseudoannulata*, which was supported by the sequence structural features, enzyme activity, and inhibitor and insecticide sensitivities. Our findings will be helpful for understanding the evolution and complexity of invertebrate AChEs.

## 4. Materials and Methods

### 4.1. Spiders, Expression Vector, Cell Lines and Chemicals

The spiders *P. pseudoannulata* were collected from paddy fields of Nanjing (Jiangsu, China) in September 2015 and stored kept at −80 °C before use. Expression vector pFastBac-HTa and Sf9 cell lines were purchased from Invitrogen (Carlsbad, CA, USA).

The chemicals eserine, 1,5-bis(4-allyldimethylammoniumphenyl)-pentan-3-one dibromide (BW284C51), tetraisopropyl pyrophosphoramide (ISO-OMPA), acetylthiocholine iodide (ATC), butyrylthiocholine iodide (BTC), propionylthiocholine iodide (PTC), dithiobis-(2-nitrobenzoic acid) (DTNB) and insecticides (fenobucarb, carbaryl and paraoxon) were purchased from Sigma (St. Louis, MO, USA). The insecticide diazoxon was purchased from J&K Scientific Ltd (Beijing, China).

### 4.2. Cloning and Homology Analysis of the Putative Ace Gene

A single female spider was used as a sample to extract the total RNA, and using 5′ and 3′ full RACE Core Set (TaKaRa, Dalian, China) to amplify the full cDNA ends according to the manufacturer’s instructions. Based on the *P. pseudoannulata* transcriptome annotation and sequence blast using NCBI online services, one putative *ace* gene was selected and acquired full sequence using RACE technology with its specific primers (Table 5). The complete amino acid sequence of the new putative AChE was aligned with other species AChEs using VectorNTI 11.5 (Invitrogen, Carlsbad, CA, USA) and GeneDoc 2.7 software (Softpedia, Romania). The phylogenetic relationships among different species AChEs were analyzed through MEGA 7.0.21 software (http://www.megasoftware.net/) using the neighbour-joining method and evaluated via bootstrapping with 1000 iterations.

### 4.3. Expression and Biochemical Properties Assaying of the Putative AChE

As described previously, complete coding sequence of the new putative *ace* gene was subcloned into the expression vector pFastBac-HTa with its specific primers (Table 5) and verified by nucleotide sequencing [12,15]. The vector with an enhanced green fluorescent protein (eGFP, GenBank accession number: AAK15492), which constructed previously in our laboratory was as a control. Using baculovirus-insect expression system, Sf9 cells which infected by the recombinant Bacmid DNAs were used for protein expression, and the baculovirus culture supernatants were collected as the crude enzyme. The culture supernatants of non-infectious Sf9 cells were used as the negative control.

The methods for enzyme biochemical properties assaying were performed as described previously [12]. Fifty microliters of enzyme, 100 μL of DTNB (75 μM) and 100 μL of substrate at different concentrations were mixed in a 96-well microplate for the enzyme activities detecting. Using Molecular Devices Thermomax Kinetic Microplate Reader, the mixed solutions were monitored for 20 min in 30 s intervals at 405 nm and three replications were used to calculate the mean level of enzyme activity. Different pH of the mixed-reaction solutions were employed for detecting enzyme optimal pH condition. Under the optimal pH of enzyme, the Michaelis–Menten constant value (*K*_M_) and maximal reaction velocity (*V*_max_) for each substrate were determined by fitting the velocity (ν) and substrate concentration ([S], 20–2000 μM) data to ν = *V*_max_ [S]/(*K*_M_ + [S]) using Prism 5.0 (GraphPad Software, La Jolla, CA, USA). Protein concentration was measured by the Protein-dye Binding Method according to the Micro BCA Protein Assay Kit (ThermoFisher, Waltham, MA, USA) instruction.

To determine *IC*_50_ values of enzyme to different inhibitors, the enzyme was first incubated with an inhibitor at various concentrations ([I]) for 10 min. The residual enzyme activity (in reference to a mixture of the enzyme and buffer) was determined as above and plotted against −log_10_[I]. The *IC*_50_ value for each inhibitor was determined from nonlinear regression analysis of activity and −log_10_[I] data using Prism 5.0 (GraphPad Software, La Jolla, CA, USA). The bimolecular rate constant (*k*_i_) was determined by enzyme reaction premixed with various concentrations of an inhibitor and was fitted using Prism 5.0.

### 4.4. Data Analysis

Pooled data are presented as the means ± SEM of at least five independent experiments. Statistical significance was determined by one-way ANOVA, and Fisher’s least significant difference *post-hoc* test was used for pair-wise comparisons. Data were considered to be significant at *p* < 0.05.

## Figures and Tables

**Table 1:** Key amino acid differences at functional sites among AChEs of *T. californica*, *T. urticae*, and *P. pseudoannulata.*

Subsite	*T. californica*	*T. urticae*	PpAChE1	PpAChE2	PpAChE3	PpAChE4	PpAChE5
Catalytic triad	S200	S	S	S	S	S	S
	E327	E	E	E	D	D	E
	H440	H	H	H	H	H	H
Oxyanion hole	G118	G	G	G	G	G	G
	G119	S	G	G	G	A	G
	A201	A	A	A	A	A	A
Choline binding site	**W84**	**W**	**W**	**W**	**W**	**W**	**W**
	**Y130**	**Y**	**Y**	**Y**	**F**	**Y**	H
	**F330**	**Y**	**Y**	**F**	T	L	**F**
	**F331**	**F**	**F**	Q	L	I	Q
Acyl pocket	**F288**	**F**	**F**	R	R	R	R
	**F290**	**F**	**F**	**F**	**Y**	T	**F**
	V400	**F**	**F**	L	R	R	L
Peripheral anionic site	**Y70**	V	I	**Y**	**Y**	**F**	**Y**
	**Y121**	**W**	**Y**	S	R	V	**F**
	**W279**	E	**W**	N	S	S	S
Wall of the gorge	**W114**	**W**	**W**	**W**	**F**	**W**	**W**
	**W233**	**W**	**W**	L	D	D	L
	**Y334**	**Y**	**Y**	T	K	N	T
	**W432**	**W**	**W**	**W**	**W**	**F**	**W**
	**Y442**	E	D	E	D	D	S

Residues in *T. californica* are used as reference values. The numbering indicates the positions of *T. californica* AChE amino acids, which starts at the N-terminus of the mature protein. Conserved aromatic residues are shown in bold type.

**Table 2:** Kinetics of substrate hydrolysis for PpAChE5.

	*K*_M_ (µM)			*V*_max_ (nmol/mg·min)			*V*_max_/*K*_M_ (mL/mg·min)			*V*_max_ Ratio		
	ATC	BTC	PTC	ATC	BTC	PTC	ATC	BTC	PTC	ATC vs. BTC	ATC vs. PTC	BTC vs. BTC
PpAChE1	536.8 ± 63.4 c	858.8 ± 103.2 c	1148.6 ± 133.5 c	112.6 ± 8.8 c	95.4 ± 12.8 b	76.2 ± 9.2 c	209.8	111.1	66.3	1.18	1.48	1.25
PpAChE2	4413.5 ± 533.7 a	4542.2 ± 326.0 a	4496.3 ± 318.8 a	253.9 ± 20.4 b	233.9 ± 16.5 a	156.8 ± 21.3 a	57.5	51.5	34.9	1.09	1.62	1.49
PpAChE3	42.9 ± 5.8 e	198.6 ± 22.3 e	503.6 ± 86.8 d	124.7 ± 11.5 c	40.3 ± 5.6 c	106.2 ± 15.1 b	2906.8	202.9	210.9	3.09	1.17	0.38
PpAChE4	61.6 ± 7.5 d	269.4 ± 35.5 d	279.3 ± 35.2 e	86.6 ± 9.7 d	105.5 ± 8.9 b	49.7 ± 8.4 d	1405.8	391.6	177.9	0.82	1.74	2.12
PpAChE5	1303.5 ± 162.9 b	1528.9 ± 118.4 b	1548.2 ± 129.1 b	428.4 ± 29.2 a	91.6 ± 11.3 b	59.0 ± 7.2 cd	328.7	59.9	38.1	4.68	7.26	1.55

Different lowercases in the same column indicate significant differences among AChEs. The data are the mean ± SEM of at least five independent experiments. Data of PpAChE1-4 were from our previous study [12]. ATC = acetylthiocholine iodide; BTC = butyrylthiocholine iodide; PTC = propionylthiocholine iodide.

**Table 3:** *IC*_50_ values for three inhibitors against enzyme activities of PpAChE5 (×10^−8^ M).

	On ATC Hydrolysis		On BTC Hydrolysis
	BW284C51	Eserine	ISO-OMPA
PpAChE1	5.12 ± 0.76 d	11.67 ± 2.06 d	>10,000
PpAChE2	254.17 ± 21.40 a	186.42 ± 25.11 a	2165.39 ± 325.67
PpAChE3	3.68 ± 0.53 e	6.52 ± 1.80 e	>10,000
PpAChE4	7.36 ± 1.05 c	24.50 ± 3.94 c	>10,000
PpAChE5	47.10 ± 6.93 b	116.28 ± 15.71 b	>10,000

Different lowercases in the same column indicate significant differences among AChEs. Data of PpAChE1-4 were from our previous study [12].

**Table 4:** Inhibition kinetics (*k*_i_) of PpAChE5 by the inhibitor eserine and four insecticides.

Compound	*k*_i_ (×10^−7^M^−1^min^−1^)
Eserine	6.24 ± 1.72 b
Fenobucarb	18.46 ± 3.85 a
Carbaryl	16.79 ± 2.90 a
Paraoxon	15.50 ± 3.32 a
Diazoxon	16.43 ± 3.58 a

Different lowercases in the same column indicate significant differences of the putative AChE. The data are the mean ± SEM of at least five independent experiments.

**Table 5:** Specific primers used for *PpAChE5* gene amplification and expression.

		Primers
RACE primers	3′outer primer	TTACAACAAGCAACCCCGACC
	3′inner primer	CCATTTCAGCGAAGCGGCATT
	5′outer primer	GGCTTCAGTCTCAACTCCAACGT
	5′inner primer	GAAGCGCCATAGCATCAACACCT
Expression primers	Sense primer:	TAGTGCGGCCGCTTTCGAATATGGCTTTTCTTTCCTTAGA
	Anti-sense primer	CTCGAGACTGCAGGCTCTAGTCAGAATCCGAAATACGGGC
